# Experimental hut evaluation of DawaPlus 3.0 LN and DawaPlus 4.0 LN treated with deltamethrin and PBO against free-flying populations of *Anopheles gambiae s*.*l*. in Vallée du Kou, Burkina Faso

**DOI:** 10.1371/journal.pone.0226191

**Published:** 2019-12-23

**Authors:** Koama Bayili, Sévérin N’Do, Rajpal S. Yadav, Moussa Namountougou, Abdoulaye Ouattara, Roch K. Dabiré, Georges A. Ouédraogo, Abdoulaye Diabaté

**Affiliations:** 1 Institut de Recherche en Sciences de la Santé/Centre Muraz, Bobo-Dioulasso, Burkina Faso; 2 Université Nazi Boni, Bobo-Dioulasso, Burkina Faso; 3 Vector Ecology and Management, Department of Control of Neglected Tropical Diseases, World Health Organization, Geneva, Switzerland; Arizona State University, UNITED STATES

## Abstract

**Background:**

In view of widespread pyrethroid resistance in malaria vectors in Africa, two long-lasting insecticidal nets (LLINs) incorporated with a synergist, piperonyl butoxide (PBO), DawaPlus 3.0 (deltamethrin + PBO in the roof panel; deltamethrin alone in the side panels) and DawaPlus 4.0 (deltamethrin + PBO in all panels), were evaluated in an experimental hut trial in a rice growing irrigated area in Burkina Faso. Efficacy of nets was tested against free-flying malaria vector, *Anopheles gambiae s*.*l*., with high pyrethroid resistance involving L1014F *kdr* and CYP6P3P450 resistance mechanisms.

**Methodology:**

The efficacy of unwashed and 20-times washed DawaPlus 3.0 (polyethylene roof panel with 120 mg/m^2^ deltamethrin and 440 mg/m^2^ PBO; polyester side panels with deltamethrin 100 mg/m^2^) and DawaPlus 4.0 (same composition as roof of DawaPlus 3.0) was evaluated against DawaPlus 2.0 (80 mg/m^2^ deltamethrin; positive control). Volunteer sleepers and treatments were rotated in huts using a Latin square design on 63 consecutive nights during August–October 2016. Mortality, human blood-feeding inhibition, deterrence and exit rates of *An*. *gambiae s*.*l*. were monitored.

**Principal findings:**

Significantly higher rates of mortality and blood-feeding inhibition were observed with unwashed DawaPlus 4.0 (36%; 47.5%) than unwashed DawaPlus 3.0 (11.8%; 33.3%), DawaPlus 2.0 (4.3%; 6.4%) or untreated net (P < 0.05). Washing reduced personal protective efficacy yet PBO-LLINs were more protective and both met the WHO criteria.

**Conclusions:**

The PBO-containing DawaPlus 4.0 significantly protected against *An*. *gambiae s*.*l*. in the study area. Unwashed DawaPlus 3.0 gave low to moderate protection against the positive control. PBO inhibits oxidase action; hence in areas with active malaria transmission having oxidase mechanisms, PBO nets could confer additional personal protection.

## Introduction

After a declining trend of malaria during 2000–2015, malaria is now showing a plateau trend with an estimated 219 million cases and 435 000 deaths worldwide in 2017 [1]. Approximately 80% of the global malaria burden is contributed by 15 countries, 14 of them located in Africa. During the past decade, large-scale implementation of long-lasting insecticidal nets (LLINs) and indoor residual spraying (IRS) has played a major role in reducing malaria morbidity and mortality in Africa south of the Sahara [2, 3]. Currently, pyrethroids only are recommended for treatment of nets owing to their long residual activity, low cost and safety [4, 5], although WHO is assessing the public health value of some innovative nets treated with a combination of a pyrethroid and either a non-pyrethroid compound or PBO.

The effectiveness of LLINs and IRS products as core malaria interventions is now threatened by insecticide resistance in malaria vectors. Since 2010, 61 countries have reported resistance of one malaria vector population to at least one class of insecticide; 50 reported vector resistance to two or more insecticide classes [6]. The success of malaria control could be jeopardized by the high intensity of insecticide resistance detected in major malaria vectors [7, 8].

In Burkina Faso, insecticide resistance in *An*. *funestus* and *An*. *gambiae s*.*l*. (consisting of *An*. *coluzzii*, *An*. *gambiae* and *An*. *arabiensis*) emerged in 1960 due to use of DDT and dieldrin [5, 9]. Insecticide resistance is widely reported in the *An*. *gambiae* species complex and has spread throughout the country [10–11]. Multi-resistant *An*. *gambiae s*.*l*. populations have also been reported [12], while pyrethroid resistance has increased 1000-fold in recent years [13]. For example, in the neighbouring country of Benin, pyrethroid resistance in *An*. *gambiae s*.*l*. has reached alarming proportions, limiting the personal protective efficacy of treated nets [14]. Therefore, there is an urgent need for the development of innovative tools to control and eliminate malaria in areas with widespread pyrethroid resistance.

Piperonyl butoxide (PBO) is a synergist that increases the toxic effects of insecticides on mosquitoes by halting their detoxification before they can reach their target site. When mosquitoes contact netting with PBO, it inhibits the action of metabolic enzymes (e.g. mixed-function oxidases), resulting in enhanced lethal effects and increased protective efficacy of these nets over pyrethroid-only LLINs [15]. WHO has recommended use of certain PBO-containing pyrethroid-LLINs in recent years following field evaluation of these products [15].

The present study evaluated the efficacy of DawaPlus 3.0 and DawaPlus 4.0 nets (manufactured by TANA Netting, United Arab Emirates) in experimental huts against wild populations of *An*. *gambiae s*.*l*. in Burkina Faso following WHOPES guidelines on LLINs [16]. The study was done in the rice growing area of Vallée du Kou in Burkina Faso having high population density of *An*. *gambiae s*.*l*. that has shown multiple mechanisms of resistance against different insecticides.

## Methods

### Study area and experimental huts

The studies were conducted at the field station in Vallée du Kou (4˚24’59” longitude west; 11˚24’ latitude), which was established in 1970. The station has an area of 1200 ha divided into seven discrete villages and has a wooded savannah climate. Mean annual rainfall is about 1100 mm, and rice is the principal crop. Insecticides are used for pest control in rice fields and extensively used in the cotton crops in surrounding villages. High densities of mosquitoes are found year-round, peaking from August to October during the rainy season. *An*. *coluzzii* predominates and is highly resistant to pyrethroids and DDT (*kdr* frequency: 0.8–0.95). Recently, ace-1 frequency enhancing [11, 12] and detoxifying enzymes have been observed in malaria vectors [17].

The study was conducted in 7 huts using a West African design to simulate local houses made of local materials surrounded by a water-filled moat to prevent entry of scavenging ants; a verandah was attached to trap mosquitoes exiting at night due either to behavioural or insecticidal effects. Mosquitoes could freely enter through four windows fitted with metal slits 1 cm apart to create an angle allowing entry but not exit. The ceilings were lined with plastic sheeting.

### Test products

- DawaPlus 3.0 LN: the side panels are made of knitted polyfilament polyester fibres of 100 denier coated with 2.5 g AI/kg deltamethrin (= 100 mg AI/m^2^); the roof panel of 130 denier polyethylene fibres incorporates, in separate fibres, 3 g AI/kg deltamethrin (= 120 mg AI/m^2^) and 11 g/kg PBO (= 440 mg/m^2^).
- DawaPlus 4.0 LN: all panels are identical to that of the roof panel of DawaPlus 3.0.
- DawaPlus 2.0 LN (positive control): a 100-denier polyester net coated with 2.0 g AI/kg deltamethrin (= 80 mg AI/m^2^).

### Treatment arms

The comparison arms tested were: (i) unwashed DawaPlus 3.0; (ii) DawaPlus 3.0 washed 20 times; (iii) unwashed DawaPlus 4.0; (iv) DawaPlus 4.0 washed 20 times; (v) unwashed DawaPlus 2.0; (vi) DawaPlus 2.0 washed 20 times; and (vii) untreated polyester net of 100 denier.

### Procedures

#### Washing of nets

The nets were washed at the laboratory of the Institut de Recherche en Sciences de la Santé (IRSS), Ouagadougou using the WHOPES Phase II washing procedure [16]. The regeneration time, i.e. the interval of time required between two consecutive washes to regenerate full efficacy, for all three nets was taken to be one day based on Phase I (laboratory) studies [16]. Nets were dried horizontally on separate mats in the shade and stored at room temperature between washes.

#### Testing in huts

The treatments were rotated weekly in huts according to the Latin square design. Three nets per treatment arm were tested each week: two nets on two nights in a given hut and the third net on three nights. The huts were cleaned at the end of each weekly round and ventilated, after which the nets were rotated to different huts. The trial was conducted for 9 consecutive weeks to collect sufficient numbers of mosquitoes for adequate statistical analysis. Before testing in huts, the nets were holed according to WHO guidelines [16] by cutting six holes of 4 cm x 4 cm each, i.e. 2 holes each in the long side panels and one hole each in the other two side panels, to simulate worn nets in routine household use.

The study was approved by the institutional ethics committee of IRSS reference no. A04-2016/CEIRES. The volunteers for the study were recruited from villages around the trial station. They were screened for malaria and given prophylaxis during their participation in the study free of cost. Written and signed consent was obtained from all seven volunteers (adult males) sleeping in huts who were recruited from villages near the study site and participated throughout the study. The volunteers were assigned randomly in the huts on the first night and thereafter they were rotated each night according to the Latin square scheme. The trial started at 20:00 each night and finished the next morning at 07:00. Before exiting the huts in the morning, the volunteer sleepers collected dead and live mosquitoes from the floor, walls, roof, verandah traps (floor, walls, roof) and inside the nets.

Mosquitoes were brought to the laboratory for species identification. Alive *An*. *gambiae s*.*l*. mosquitoes were held in plastic cups and provided access to 10% glucose solution soaked in cotton wool; the cups were stored in the laboratory at 27 ± 2 ˚C and 80 ± 10% relative humidity. Delayed mortality was scored after 24 h of collection.

### Efficacy criteria

Mosquitoes were scored as dead or alive and as blood-fed or unfed. The outcomes measured were: (i) deterrence, i.e. reduction in mosquito entry in huts with treated nets relative to the control huts (untreated nets); (ii) induced exophily, i.e. the proportion of mosquitoes collected in the verandah trap relative to the total number collected in the hut; (iii) blood-feeding inhibition, i.e. proportional reduction in blood-feeding of mosquitoes in treated huts relative to the control huts; (iv) immediate and delayed mortality, i.e. the proportion of dead mosquitoes when collected in the morning and after 24 h of holding in the laboratory relative to the total number collected in huts, respectively.

Deterrence and blood-feeding inhibition are personal protection rate indicators, which are calculated by the following formula [16]:

$$Personal\;protection\;rate\;\left(\%\right)=100\;\left(B_{u}-B_{t}\right)/B_{u},$$

where B_u_ is the total number of blood-fed mosquitoes in the huts with untreated nets and B_t_ is the total number of blood-fed mosquitoes in the huts with treated nets.

The killing effect of each treatment was estimated as follows [16]:

$$Killing\;\left(insecticidal\right)\;effect\;\left(\%\right)=100\;(K_{t}-K_{u})/T_{u},$$

where K_t_ is the number of mosquitoes killed in the huts with treated nets, K_u_ is the number of mosquitoes found dead in the huts with untreated nets, and T_u_ is the total number of mosquitoes collected from the huts with untreated nets.

According to the WHOPES criteria [16], the efficacy criteria for candidate nets washed 20 times is that they should perform equal to or better than the positive control net, i.e. 20-times washed DawaPlus 2.0 LN. WHO has set 20 washes as the estimated number of washings during the effective life of three years of a net taking 4 washes per year.

### Bioassays on nets in situ against *An*. *gambiae s*.*l*

In this procedure, mortality of adult *An*. *gambiae s*.*l*. that emerged from larvae collected from the natural habitats in the study area during August–October 2016 was tested by fixing cones on nets in situ. Laboratory-reared *An*. *gambiae* Kisumu (fully susceptible) strain was used as a control. The cone bioassays were done against nets before and after the hut trial. Ten cones were fixed on a net from each treatment arm: for DawaPlus 3.0, five cones were fixed on the roof panel and five cones on the side panels; for the other nets, two cones were fixed on each of the five panels. Ten 3–5-day old, non-blood fed female mosquitoes were introduced in each cone for 3 min to allow contact on the netting. A total of 100 mosquitoes were tested per net. After exposure, mosquitoes were transferred to holding cups and provided access to sugar solution. Knock down was scored 60 min after exposure and mortality 24 h after exposure.

### Tunnel test

The tunnel test enables measurement of mortality and the blood-feeding success of host-seeking mosquitoes in tunnel equipment. Although tunnel tests are not required in Phase II studies according to WHOPES guidelines, they provide further insight into the protective efficacy of unwashed and washed nets. The tunnel tests were done on the unwashed and 20-times washed nets using netting pieces from the roof and the side panels of the fourth net from all treatment arms using *An*. *gambiae* Kisumu strain and *An*. *gambiae s*.*l*. adults emerged from larvae collected from the study area.

The tunnel test procedure followed WHO guidelines [16] and was replicated three times. A guinea pig was placed in the smallest chamber of the tunnel as a bait and batches of non-blood fed 4–7-day old female mosquitoes were released into the biggest chamber at 18:00. Holed pieces of net were fixed in the tunnel and mosquitoes could fly freely to make contact with the net sample before penetrating the holes to the baited compartment. The next morning at 08:00, the mosquitoes were collected from each section of the tunnel separately and counted as dead, alive, blood-fed and non-blood fed. Surviving females were held in paper cups, provided access to sugar solution and delayed mortality was recorded 24 h later.

The protective efficacy of nets tested was measured in terms of blood-feeding inhibition; immediate and delayed mortality, and the repellent effect (proportion of mosquitoes penetrating the holes in the netting piece).

### Insecticide susceptibility and synergistic effect of PBO

Susceptibility of the *An*. *gambiae* Kisumu strain maintained in the insectary and of the adults emerged from the field-collected *An*. *gambiae s*.*l*. larvae was tested according to the WHO procedure using papers impregnated with permethrin (0.75%) and deltamethrin (0.05%) [18].

The synergistic effect of PBO on the mortality of these mosquito strains was also tested using the CDC bottle bioassays [19]. Batches of mosquitoes were separately exposed in 250 mL bottles as follows: permethrin (21.5 μg/bottle) for 60 min; PBO-treated bottles (400 μg/bottle) for 60 min followed by permethrin-treated bottles for another 60 min; and bottles treated with permethrin and PBO. After exposure, mosquitoes were aspirated from bottles and held in paper cups for 24 h to record delayed mortality. Four to six replicates of each treatment and a similar number of the control (acetone only) were performed.

### Determination of chemical content in netting samples

Pieces of nettings were cut before and after washing nets in different treatment arms and at the end of their testing in huts [16] for analysis of deltamethrin and PBO content at the WHO Collaborating Centre (Gembloux, Belgium; http://www.cra.wallonie.be) using analytical methods developed by the Collaborative International Pesticides Analytical Council (CIPAC; https://www.cipac.org/). Deltamethrin content was determined in samples of DawaPlus 3.0 side panels and DawaPlus 2.0 using the standard CIPAC method 333/LN/(M)/3; the same in samples of DawaPlus 3.0 roof, DawaPlus 4.0 and control (untreated) nets was analysed using CIPAC method 333/LN/(M2)/3. PBO content in DawaPlus 3.0 roof, DawaPlus 4.0 and untreated nets was analysed using CIPAC methods 333/LN/(M2)/3 for the extraction and 33/LN/(M)/3 for the chromatographic determination.

### Statistical analysis

The data were recorded in Excel 2013 and analyzed using R software. Experimental trial data (mortality rate, blood-feeding inhibition, deterrency and exophily) were analysed using the Kruskal-Wallis test through Dunn’s Multiple Comparison Test, pairwise comparisons were performed to compare treatments. Proportional exiting of mosquitoes in the verandah trap was analysed using one-way ANOVA through Tukey’s Multiple Comparison Test to compare all the treatments. Outcomes of experimental hut trial (net penetration, blood-feeding, exiting and mortality) of each treatment were assessed using binomial generalized linear mixed models. A logit link function was fitted using the “lme4” package for R software (version 2.15.0) (https://www.r-project.org/).

## Results

### Insecticide susceptibility of colonized and wild population of mosquitoes

The laboratory colonized *An*. *gambiae* Kisumu strain was found to be fully susceptible to permethrin (0.75%) and deltamethrin (0.05%), but *An*. *gambiae s*.*l*. (wild population) was highly resistant to both insecticides (mortality < 8%) using WHO impregnated papers (Table 1). In CDC bottle assays, mortality induced with permethrin alone and permethrin plus PBO using *An*. *gambiae* Kisumu was 100% (Table 2). *Anopheles gambiae s*.*l*. was found resistant to permethrin (mortality about 27%), but upon exposure to PBO followed by exposure to permethrin, or exposure to their mixture in bottles, mortality increased up to 80% (Table 2). These results showed high prevalence of oxidases in this population. In recent studies in the Vallée du Kou, specific responsible genes have been identified such as non-detoxification genes, e.g. cell transporters and cuticular components [17].

**Table 1:** Results of WHO tube test with 60 min exposure of *An*. *gambiae* Kisumu and *An*. *gambiae s*.*l*. (emerged from field-collected larvae) on WHO impregnated papers.

Treatment	*An*. *gambiae* Kisumu			*An*. *gambiae s*.*l*.		
	Number tested	% knock down	% mortality	Number tested	% knock down	% mortality
**Control**	50	0	0	53	0	2
**Permethrin 0.75%**	110	100	100	109	12	7
**Deltamethrin 0.05%**	102	100	100	98	14	6

**Table 2:** Knock down and mortality rates of *An*. *gambiae* Kisumu and *An*. *gambiae s*.*l*. (emerged from field-collected larvae) in CDC bottle bioassays using PBO as a synergist.

Treatment	*An*. *gambiae* Kisumu			*An*. *gambiae s*.*l*.		
	Number tested	% knock down	% mortality	Number tested	% knock down	% mortality
**Control (acetone only)**	52	4	6	49	0	0
**PBO (400 μg)**	45	0	4	51	0	2
**Permethrin (21.5 μg)**	52	100	100	51	80	27
**PBO (400μg) + Permethrin (21.5 μg)**	51	100	100	51	80	88
**PBO followed by Permethrin**	55	100	100	50	82	92

### Experimental hut trial

A total of 11389 *An*. *gambiae s*.*l*. specimens were collected from all huts during the 9-weeks study.

#### Mosquito mortality

The unwashed DawaPlus 4.0 scored the highest 24 h of wild, free-flying *An*. *gambiae s*.*l*. (43%), followed by the unwashed DawaPlus 3.0 (21.7%) and unwashed DawaPlus 2.0, the positive control (15%) (Table 3). The mortality was significantly higher with the unwashed DawaPlus 4.0 than the other treatments (P < 0.05 using Tukey Multiple Comparison Test). DawaPlus 3.0 produced low mortality but it was significantly higher than unwashed and 20-times washed DawaPlus 2.0 (P < 0.05). There was no significant difference in mortality of unwashed or 20-times washed DawaPlus 2.0, 20-times washed DawaPlus 3.0 and 20-times washed DawaPlus 4.0 (P > 0.05).

**Table 3:** Mortality, blood-feeding and blood-feeding inhibition rates in wild, free-flying *An*. *gambiae s*.*l*. mosquitoes.

Parameters	Untreated net	Unwashed DawaPlus 2.0	DawaPlus 2.0 washed 20 times	Unwashed DawaPlus 3.0	DawaPlus 3.0 washed 20 times	Unwashed DawaPlus 4.0	DawaPlus 4.0 washed 20 times
**Mortality** **(%) and** **95% CI)**	11.2^a^ (5.8–13.6)	15.0^a^^,^^c^ (13.7–20.2)	16.7^a^^,^^c^ (12.1–21.0)	21.7^b^^,^^c^ (14.2–27.7)	17.5^a^^,^^c^ (13.7–19.2)	43.1^b^*** (39.2–47.7)	15.4^a^^,^^c^ (13.7–22.3)
**Corrected mortality (%**		4.3	6.1	11.8	7.1	36	4.7
**Blood-feeding rate** **(%) and 95% CI)**	64.4^a^ (54.8–80.2)	60.3^a^^,^^c^ (50.5–81.0)	50.3^a^^,^^c^^,^^d^ (46.6–64.0)	42.93^b^^,^^c^^,^^d^ (36.2–59.2)	45.5^a^^,^^c^^,^^d^ (41.7–61.8)	33.8^b^ (21.9–45.5)	40.6^b^^,^^d^ (34.9–56.9)
**Blood-feeding** **inhibition rate (%)**	–	6.4^a^	21.9^a^^,^^b^	33.3^b^	29.4^b^	47.5^c^	36.9^b^^,^^c^

a, b, c, d: The values labelled with the same letter per row are not significantly different (P > 0.05).

*** high significant difference (P<0.0001). CI: confidence intervals

#### Blood-feeding inhibition

Significantly low blood-feeding was observed with unwashed DawaPlus 4.0 than with unwashed DawaPlus 2.0 as a positive control (P = 0.0067). Relative to the untreated net, there was high blood-feeding inhibition with unwashed (47.5%) and 20-times washed (36.9%) DawaPlus 4.0 (Table 3). Blood-feeding inhibition with unwashed and 20-times washed DawaPlus 3.0 was 33.3% and 29.4%, respectively, compared with DawaPlus 2.0 (6.4%; 21.9%). There was no significant difference between unwashed and 20-times washed DawaPlus 3.0 or between unwashed and 20-times washed DawaPlus 4.0 (P > 0.05).

#### Mosquito entry in huts and exiting to verandah trap

An average of 1627 female *An*. *gambiae s*.*l*. were collected per treatment during the 9-week trial. The deterrence rate was highest for DawaPlus 4.0 followed by DawaPlus 3.0 and DawaPlus 2.0 (Table 4). Deterrence is the reduction in mosquito entry in huts with treated nets relative to the control huts with untreated nets.

**Table 4:** Deterrence, exiting rate and insecticide induced exiting rate of *An*. *gambiae s*.*l*. in different treatment arms in the experimental hut trial.

Treatments	Number of washes	Number of mosquitoes caught	Deterrence %	Exiting rate % (95% confidential interval)	Induced exiting rate %
**Untreated control**	0	1848	–	29.32^a^ (23.21–32.75)	–
**DawaPlus 2.0**	0	1548	16.23	40.37^b^ (34.64–53.32)	27.35
	20	2155	0	48.21^b^^,^^c^ (44.34–60.15)	39.16
**DawaPlus 3.0**	0	1365	26.13	50.84^b^^,^^c^ (40.70–63.74)	42.31
	20	1981	0	47.95^b^ (41.79–57.01)	38.84
**DawaPlus 4.0**	0	846	54.22	69.73^c^ (61.00–75.24)	57.94
	20	1646	10.93	50.36^b^ (41.03–61.78)	41.76

a, b, c: Values sharing the same superscript letter in the column are not significantly different at the 5% level.

Exiting into the verandah trap was significantly high (P < 0.0001) for washed and unwashed treated nets (40–70%) compared with untreated nets, and insecticide induced exiting rates relative to untreated nets ranged from 27% to 58% with all treatments. Induced exophily is the proportion of mosquitoes collected in the verandah trap relative to the total number collected in the hut.

#### In situ cone bioassays

**Fig 1:**
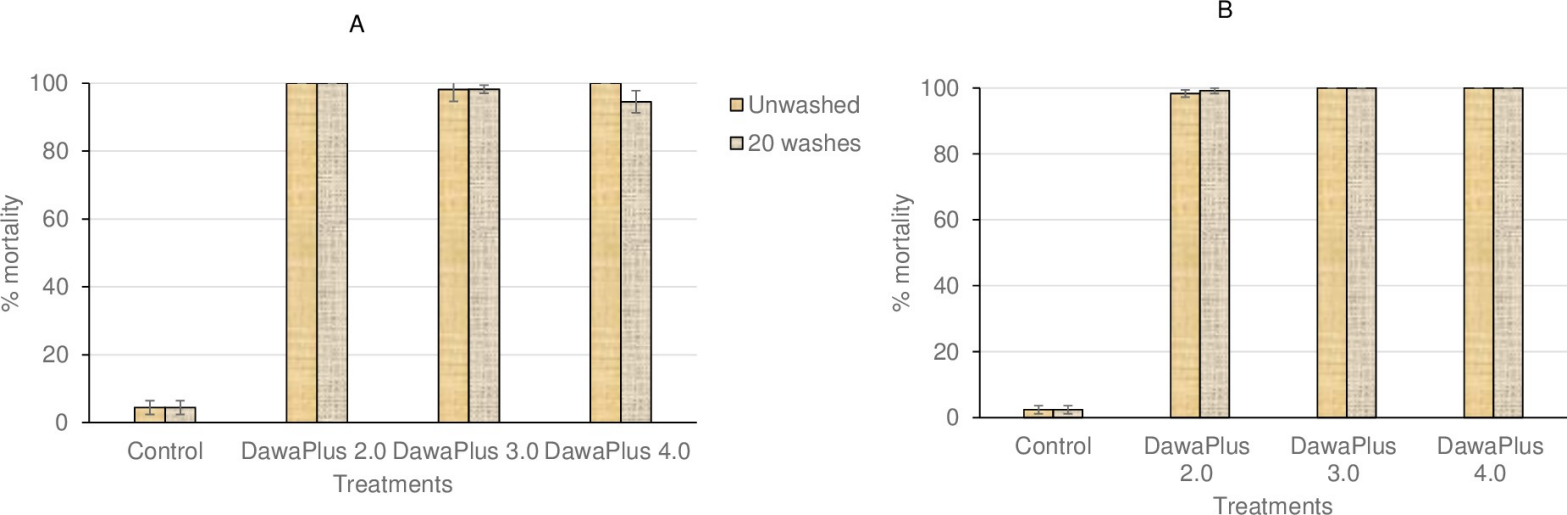
Mortality of *An*. *gambiae* Kisumu strain performed in situ on the nets using WHO cones. **** Bars show mortality rates at the beginning of the trial (A) and at the end (B) of the trial with unwashed and 20-times washed nets. Error bars represent 95% confidence intervals.

**Fig 2:**
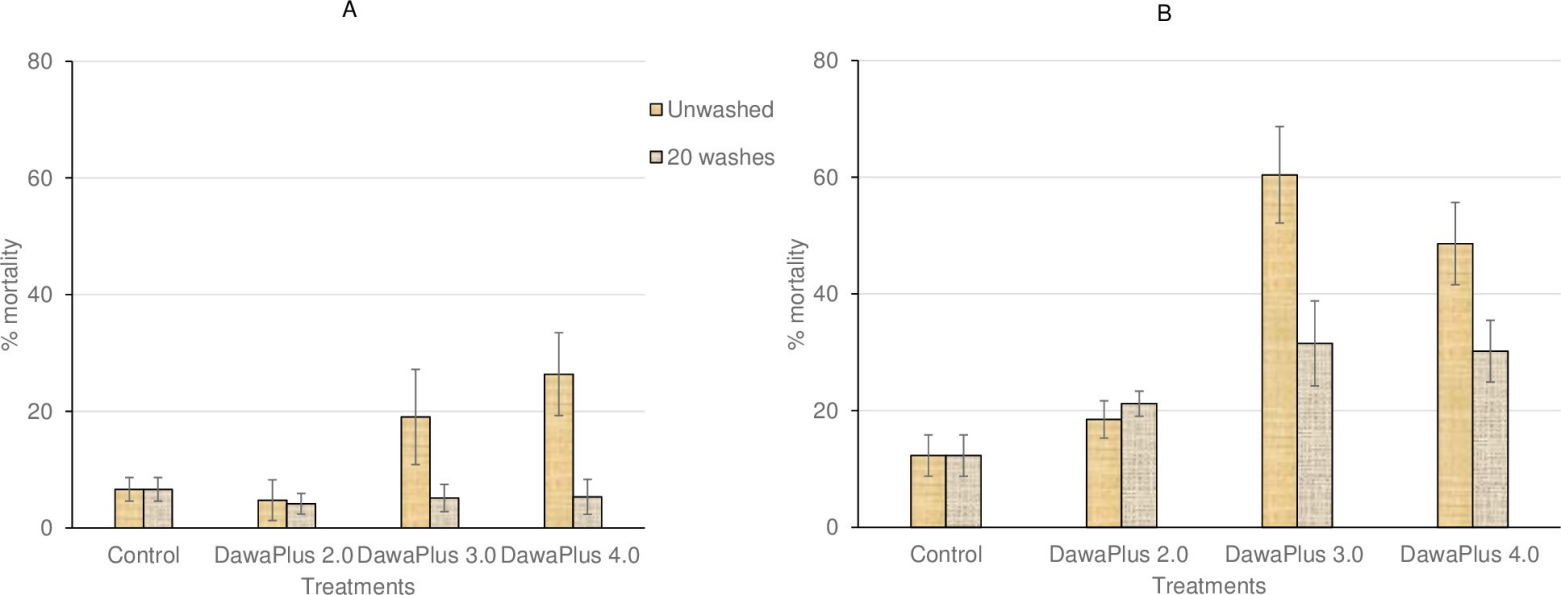
Mortality of *An*. *gambiae s*.*l*. adults emerged from field collected larvae in Vallée du Kou in cone bioassays performed in situ on the nets using WHO cones. Bars show mortality rates at the beginning of the trial (A) and at the end of the trial (B) with unwashed and 20-times washed nets. Error bars represent 95% confidence intervals.

In cone bioassays in situ on nets, the mortality in *An*. *gambiae* Kisumu (susceptible strain) was < 5% with untreated net and 100% with treated nets at the beginning (Fig 1A) and end of the trial (Fig 1B). Using wild strain (*An*. *gambiae s*.*l*.), all three treated nets caused < 30% mortality at the beginning of the trial (Fig 2A), although at the end of the trial, unwashed DawaPlus 3.0 and 4.0 showed 60% and 50% mortality respectively, while other treatments still showed low mortality (< 30%) (Fig 2B).

### Tunnel test

**Fig 3:**
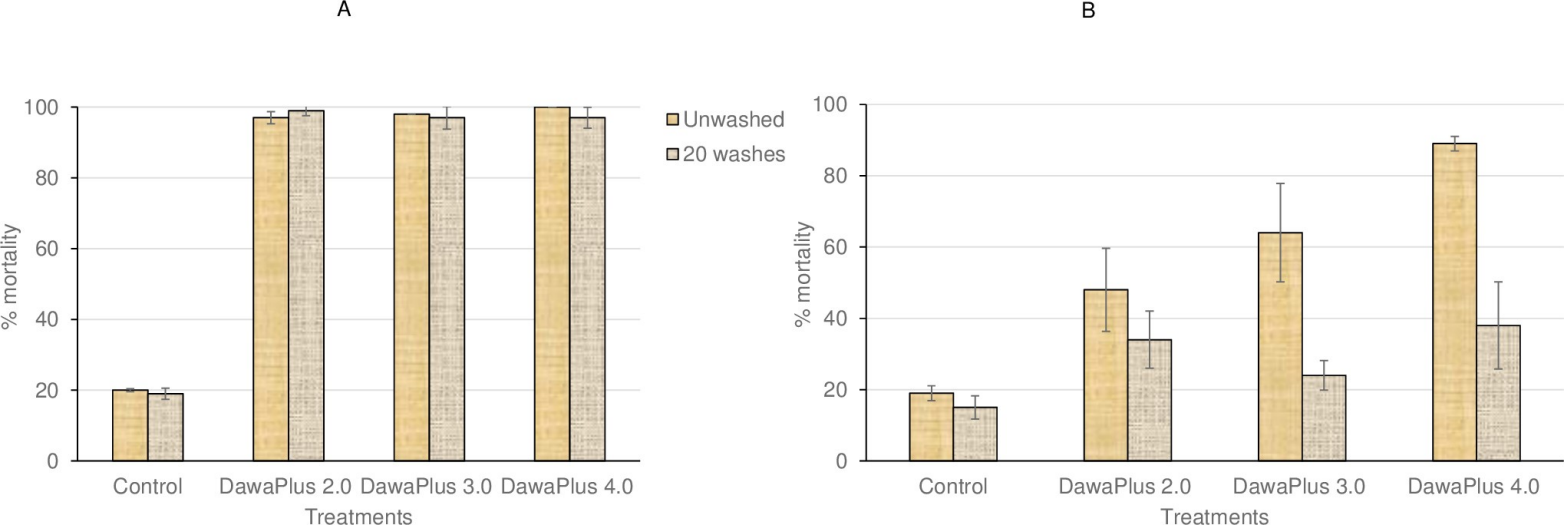
**Mortality of adults of *An*. *gambiae* Kisumu (A) and *An*. *gambiae s*.*l*. (emerged from field-collected larvae) (B) in tunnel tests with different treatments.** Bars show mortality rates of unwashed and 20-times washed nets in different treatments. Error bars represent 95% confidence intervals.

The treated nets caused 100% mortality in *An*. *gambiae* Kisumu strain (Fig 3A). Mortality in *An*. *gambiae s*.*l*. emerged from field-collected larvae was as follows: unwashed DawaPlus 4.0 (89%); unwashed DawaPlus 3.0 (64%); and unwashed DawaPlus 2.0 (48%). After 20-times washing of nets, mosquito mortality dropped to 40% in all treatments (Fig 3B).

**Fig 4:**
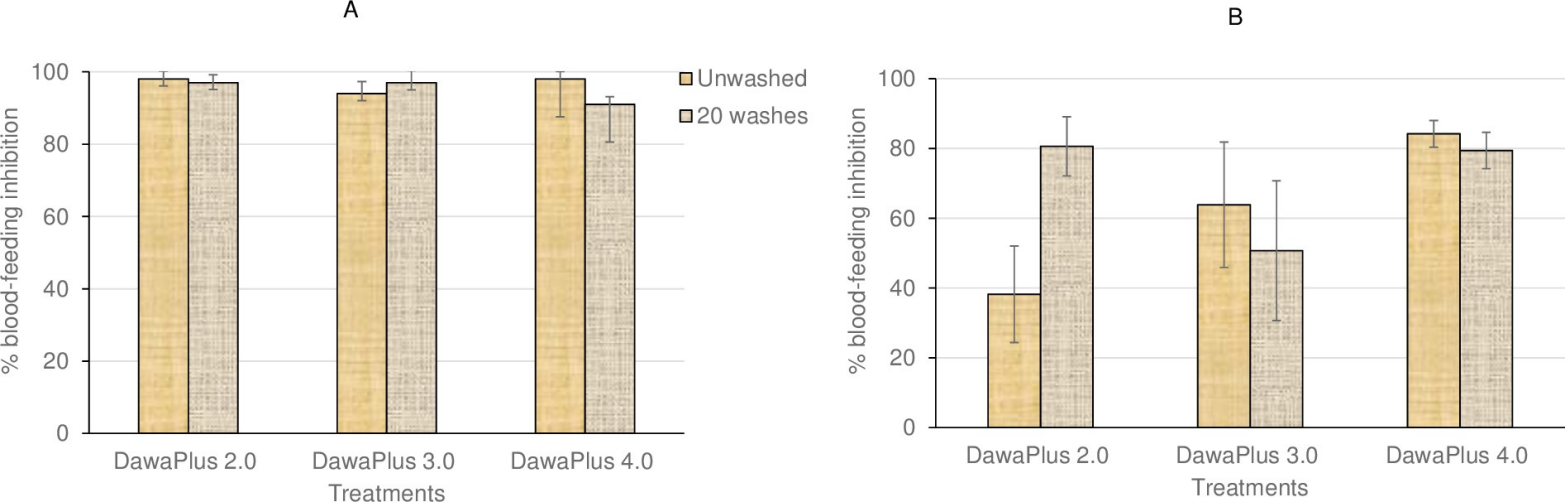
**Blood-feeding inhibition rate of *An*. *gambiae* Kisumu (A) and *An*. *gambiae s*.*l*. (emerged from field-collected larvae) (B) in tunnel tests with different treatments relative to the untreated control.** The bars show blood-feeding inhibition rate of unwashed and 20-times washed nets. Error bars represent 95% confidence intervals.

All the LLINs completely inhibited blood-feeding of the susceptible Kisumu strain (Fig 4A). Blood-feeding inhibition for other nets was as follows: unwashed and 20-times washed DawaPlus 4.0 up 80% with resistant strain (Fig 4B); unwashed and 20-times washed DawaPlus 3.0 > 60% and 46%, respectively; unwashed DawaPlus 2.0 < 40%, and DawaPlus 2.0 washed 20-times 80%.

Compared with the untreated control net, fewer *An*. *gambiae* Kisumu penetrated the holes in treated nettings in tunnel tests except for the unwashed DawaPlus 3.0 (Table 5). However, a greater number of *An*. *gambiae s*.*l*. penetrated the holes in treated nettings, which may be due to pyrethroid resistance in the wild vector population.

**Table 5:** Rate of penetration through holes and blood-feeding rate of *An*. *gambiae* (Kisumu strain) and *An*. *gambiae s*.*l*. (wild strain emerged from field-collected larvae) in tunnel tests.

Treatment	Number of washes	*An*. *gambiae* Kisumu			*An*. *gambiae s*.*l*.		
		Number tested	% passing	% blood- feeding	Number tested	% passing	% blood- feeding
**Control (untreated net)**	0	158	85.44	80.37	281	64.76	59.78
	20	185	76.75	60	281	64.05	59.78
**DawaPlus 2.0 (roof and side panels)**	0	188	14.36	1.59	314	51.27	36.94
	20	209	18.66	1.43	319	25.7	11.59
**DawaPlus 3.0 (roof and side panels)**	0	212	56.6	4.24	287	42.5	21.6
	20	177	22.03	1.69	346	39.01	29.47
**DawaPlus 4.0 (roof and side panels)**	0	237	29.11	1.68	328	35.67	9.45
	20	196	25	5.1	317	34.38	12.3

### Chemical content

The deltamethrin and PBO content in unwashed and 20-times washed nets used before and after the trial are given in Table 6. The deltamethrin content of unwashed nets was within ± 25% of the target dose according to the tolerance limit given in the WHO specification of the products [16].

The deltamethrin retention rate after 20-times washing of nets was 35%, 8% and 60% respectively for DawaPlus 2.0, DawaPlus 3.0 and DawaPlus 4.0 (Table 6), which further declined after the trial (Table 6). The PBO content in unwashed nets was within ± 25% of the target dose. It decreased after washing in the DawaPlus 3.0 roof from 8.7 to 5.7 g/kg and in the DawaPlus 4.0 from 10.4 to 2.4 g/kg.

**Table 6:** Content of deltamethrin active ingredient (AI) and piperonyl butoxide (PBO) and their retention rate in treated nets.

Treatment	Number of washes	AI content before trial (g/kg)	AI content after trial (g/kg)	Target concentration (g/kg)	Retention (%) after washing
**Deltamethrin AI content**					
**DawaPlus 2.0**	0	2.2	1.94	2	–
	20	0.77	0.79	2	35
**DawaPlus 3.0 sides**	0	2.64	2.44	2.5	–
	20	0.22	0.19	2.5	8
**DawaPlus 4.0**	0	3.11	2.46	3	–
	20	1.87	2.14	3	60
**PBO content**					
**DawaPlus 3.0 roof**	0	8.7	6.9	11	–
	20	5.7	4.3	11	65
**DawaPlus 4.0**	0	10.4	9.1	11	–
	20	2.4	1.8	11	23

## Discussion

In recent years, resistance in malaria vectors to insecticides, particularly to pyrethroids, has spatially expanded and intensified, causing technical problems in malaria control. The number of mosquito species developing resistance to insecticides is increasing. Currently, LLINs and IRS are the principal vector control tools against malaria and WHO recommends their use as the core interventions [5]. Currently, six classes of insecticides are used in products recommended by WHO for adult mosquito control. Greater investments are being made in developing new vector control tools, although only one new insecticide repurposed from agriculture (i.e. clothianidin) has been introduced in public health market after several decades. It is therefore essential to maintain or restore the effectiveness of existing products/formulations to control malaria by effectively managing insecticide resistance. The use of nets treated with a mixture of insecticides having unrelated mode of action such as in Interceptor G2 nets [20] or the use of nets treated with a pyrethroid-synergist combination have shown significant impact on pyrethroid resistant mosquitoes [15, 21].

The objective of this study was to evaluate in the context of a field trial using experimental hut bioassay techniques the effectiveness and wash resistance of two pyrethroid nets containing PBO, i.e. DawaPlus 3.0 and DawaPlus 4.0. PBO has been used to increase the effectiveness of some insecticides. This improvement stems from its proficiency to inhibit two major metabolic enzyme systems, P450s and non-specific esterases, and to improve the cuticular penetration of the insecticide [22].

Experimental hut trial is a standard technique recommended by WHO to test the effectiveness of LLINs. In the present trial, mortality of the host-seeking *An*. *gambiae s*.*l*. in huts with pyrethroid-only DawaPlus 2.0 LN was < 20%. This narrow mortality response is a characteristic of other pyrethroid-only LLINs evaluated in Burkina Faso [13] where the malaria vector *An*. *gambiae s*.*l*. has achieved high-level resistance to pyrethroids through a combination of L1014F *kdr* and CYP6P3P450 mechanisms, although other metabolic enzymes such as carboxylesterases (COEAE3G, COEAE4G) and a GST (GSTE5) contribute to the problem [17].

*An*. *gambiae s*.*l*. Kisumu strain was found fully susceptible (100% mortality) to papers impregnated with deltamethrin and permethrin as well as to all treatments in the cone tests and tunnel tests, whereas the wild strain showed a high level of resistance.

DawaPlus 3.0 with PBO in its roof panel only gave no better protection than the pyrethroid-only DawaPlus 2.0 (p = 0.73). This could be due to a low contact of mosquitoes with the PBO incorporated roof panel in DawaPlus 3.0.

The unwashed DawaPlus 4.0 with deltamethrin and PBO incorporated in separate knitted fibres inhibited blood-feeding by 47.5% and killed 43% of host-seeking wild *An*. *gambiae s*.*l*. Similar results were reported by another study with PermaNet 3.0 nets [13]. An additional 31.5% mortality caused by unwashed DawaPlus 4.0 beyond that caused by unwashed DawaPlus 2.0 could be due to the synergistic effect of PBO with deltamethrin as well as significantly higher content of deltamethrin in DawaPlus 4.0 compared to DawaPlus 2.0. Earlier studies have shown that PBO increases the effectiveness of pyrethroid insecticides in resistant mosquito populations [15, 23]. Some innovative nets have combined pyrethroid treatment with the synergist PBO to overcome the P450-based metabolic resistance to pyrethroids in mosquitoes [24].

Higher mortality in *An*. *gambiae s*.*l*. with nets containing PBO was also observed in WHO cone tests in situ 24 hours post-exposure compared with DawaPlus 2.0 that had caused low mortality rates. Similar results were observed in tunnel test with unwashed nets. After 20 washes, all the nets gave decreased efficacy as confirmed by chemical analysis results.

Unwashed DawaPlus 4.0 caused high mosquito mortality in huts. The contribution of DawaPlus 4.0 to personal protection and blood-feeding inhibition compared with DawaPlus 2.0 was > 20%. Higher deterrence and exiting effects of unwashed DawaPlus 4.0 than DawaPlus 2.0 indicated its increased toxicity against mosquitoes due apparently to the presence of PBO. These results were also confirmed in the tunnel tests at the laboratory. Although net with the combination of deltamethrin and PBO showed best efficacy than the reference net, entomological parameters stayed low. It remains also unknown if the escalation of resistance is associated with overexpression of metabolic resistance genes such as *CYP6P9a/b* and if such increased expression of CYP genes could reduce the inhibition effect of PBO and reduce the efficacy of PBO-based nets [25].

There was no significant difference in mortality, blood-feeding inhibition and insecticide induced exiting rates caused by unwashed DawaPlus 2.0 (positive control) and DawaPlus 3.0. Similar results were obtained with washed DawaPlus 2.0 and DawaPlus 3.0 and 4.0.

Widespread pyrethroid resistance in mosquito vectors has resulted in reduced protective efficacy of pyrethroid-treated LLINs. In pyrethroid-resistance areas, experimental hut trials have shown substantially reduced mosquito mortality rates and protection from blood-feeding [25]. The use of pyrethroid-PBO combination nets has the potential of restoring their mosquito killing effect in areas where the resistance is due to P450 and oxidases mechanisms only |26]. Evaluation of pyrethroid-PBO nets in experimental hut trials across Africa has shown higher rates of mosquito mortality and blood-feeding inhibition than the conventional pyrethroid-LLINs, although these effects were found to be variable [26].

## Conclusions

The Phase II (experimental hut) study confirmed that DawaPlus 3.0 LN and DawaPlus 4.0 LN met the WHOPES Phase II efficacy requirements for LLINs [16]. Our data also suggest that DawaPlus 4.0 LN provides additional benefit over pyrethroid-only LLIN (reference LLN). The wild population of *An*. *gambiae s*.*l*. was highly resistant to pyrethroids, which could be due to involvement of multiple pyrethroid resistance mechanisms in the study area.

Pyrethroid-LLINs incorporated with PBO where it can be bioavailable up to at least 20 washes could provide significantly higher and longer protective efficacy in regions of moderate-to-high pyrethroid resistance. They could also be effective in areas where resistance to oxidase mechanisms (i.e. where PBO can significantly increase susceptibility in bioassays) predominates and where malaria prevalence is high. In the United Republic of Tanzania, efficacy of 33% was reported in a recent study with PBO LLINs compared with conventional LLINs after 2 years of their routine use in households [21]. In 2018, WHO recommended that national malaria control programmes consider use of pyrethroid nets with PBO in areas with pyrethroid resistance [6]. The low efficacy of pyrethroid-PBO nets in the current study in Vallée du Kou (Burkina Faso) therefore highlights an urgent need to investigate the underlying causes of low efficacy of PBO nets.

Elimination of malaria requires increased access and high coverage of at-risk populations to quality-assured, effective vector control interventions. In the face of increasing insecticide resistance, there is a need for cost-effective technologies and new tools that can restore the efficacy of available tools.

## Supporting information

S1 File Summary of hut trial data. (XLSX)

S2 File Results of World Health Organization in situ cone bioassay using *An*. *gambiae* Kisumu and a wild resistant strain (VK) on the treated nets. (XLSX)

S3 File Results of tunnel bioassay test using *An*. *gambiae* Kisumu and a wild resistant strain (VK) on treated nets from the experimental hut trial. (XLSX)

S4 File Effect of PBO on mosquitoes mortality in CDC bottle assays using *An*. *gambiae* Kisumu and a wild resistant strain (VK). (XLSX)

S5 File Report of chemical content in nets analyzed by the HPLC method. (PDF)

S6 File R analyzed scripts and tests. (R)

## Abbreviations

AI: Active ingredient
An: Anopheles
ANOVA: Analysis of variance
CDC: United States Centers for Disease Control and Prevention
CEIRES: Comité d’Ethique Institutionnel pour la Recherche en Sciences de la Santé
CIPAC: Collaborative International Pesticides Analytical Council
DDT: dichloro-diphenyl-trichloroethane
GC: Gembloux centre
GLMM: general linear mixed models
IRS: indoor residual spraying
IRSS: Institut de Recherche en Science de la Santé
KD: knock down
kdr: knock down resistance
LLIN: long-lasting insecticidal net
PBO: piperonyl butoxide
s.l.: sensu lato
WHO: World Health Organization
WHOPES: WHO Pesticide Evaluation Scheme

## References

[1] WHO. World malaria report 2018. Geneva: World Health Organization; 2018 Available from: http://www.who.int/iris/handle/10665/275867.

[2] WHO. World malaria report 2015. Geneva: World Health Organization; 2015 Available from: https://apps.who.int/iris/bitstream/handle/10665/200018/9789241565158_eng.pdf.

[3] Protopopoff N, Wright A, West PA, Tigererwa R, Mosha FW, Kisinza W, et al Combination of insecticide treated nets and indoor residual spraying in northern Tanzania provides additional reduction in vector population density and malaria transmission rates compared to insecticide treated nets alone: A randomised control trial. PLoS One. 2015;10:1–11. 10.1371/journal.pone.0142671 PMC4646432 26569492

[4] Zaim M, Aitio A, Nakashima N. Safety of pyrethroid-treated mosquito nets. Med Vet Entomol. 2000 14(1):1–5. 10.1046/j.1365-2915.2000.00211.x 10759305

[5] WHO. Global plan for insecticide resustance management in malaria vectors. Geneva: World Health Organization, 2012 Available from: https://apps.who.int/iris/bitstream/handle/10665/44846/9789241564472_eng.pdf.

[6] WHO. World malaria report 2017. Geneva: World Health Organization; 2018 Available from: https://apps.who.int/iris/bitstream/handle/10665/259492/9789241565523-eng.pdf.

[7] Kelly-Hope L, Ranson H, Hemingway J. Lessons from the past: managing insecticide resistance in malaria control and eradication programmes. Lancet Infect. Dis. 2008;8:387–389. 10.1016/S1473-3099(08)70045-8 18374633

[8] Hemingway J, Ranson H, Magill A, Kolaczinski J, Fornadel C, Gimnig J, et al Averting a malaria disaster: will insecticide resistance derail malaria control? Lancet 2016;387(10029):1785–1788. 10.1016/S0140-6736(15)00417-1 26880124 PMC6215693

[9] Asidi AN, N’Guessan R, Hutchinson RA, Traoré-Lamizana M, Carnevale P, Curtis CF. Experimental hut comparisons of nets treated with carbamate or pyrethroid insecticides, washed or unwashed, against pyrethroid-resistant mosquitoes. Med Vet Entomol. 2004;18:134–140. 10.1111/j.0269-283X.2004.00485.x 15189238

[10] Diabaté A, Baldet T, Chandre F, Guiguemdé RT, Brengues C, Guillet P, et al First report of the kdr mutation in *Anopheles gambiae* M form from Burkina Faso, West Africa. Parassitologia. 2002;44:157–158. 12701378

[11] Dabiré KR, Diabaté A, Namontougou M, Djogbenou L, Kengne P, Simard F, et al Distribution of insensitive acetylcholinesterase (ace-1R) in *Anopheles gambiae* s.l. populations from Burkina Faso (West Africa). Trop Med Int Health. 2009;14:396–403. 10.1111/j.1365-3156.2009.02243.x 19254231

[12] Namountougou M, Simard F, Baldet T, Diabaté A, Ouédraogo JB, Martin T, et al Multiple insecticide resistance in *Anopheles gambiae* s.l. populations from Burkina Faso, West Africa. PLoS One. 2012;7:e48412 10.1371/journal.pone.0048412 23189131 PMC3506617

[13] Toé KH, Jones CM, N’fale S, Ismai HM, Dabiré RK, Ranson H. Increased pyrethroid resistance in malaria vectors and decreased bed net effectiveness, Burkina Faso. Emerg Infect Dis. 2014;20:1691–1696. 10.3201/eid2010.140619 25279965 PMC4193182

[14] N’Guessan R, Boko P, Odjo A, Akogbéto M, Yates A, Rowland M. Chlorfenapyr: a pyrrole insecticide for the control of pyrethroid or DDT resistant *Anopheles gambiae* (Diptera: Culicidae) mosquitoes. Acta Trop. 2007;102:69–78. 10.1016/j.actatropica.2007.03.003 17466253

[15] WHO. Conditions for use of long-lasting insecticidal nets treated with a pyrethroid and piperonyl butoxide. Geneva: World Health Organization; 2015 Available from: https://www.who.int/malaria/areas/vector_control/use-of-pbo-treated-llins-report-nov2015.pdf.

[16] WHO. Guidelines for laboratory and field-testing of long-lasting insecticidal nets. Geneva: World Health Organization; 2013 Available from: https://apps.who.int/iris/bitstream/handle/10665/80270/9789241505277_eng.pdf.

[17] Toé KH, N’Falé S, Dabiré RK, Ranson H, Jones CM. The recent escalation in strength of pyrethroid resistance in *Anopheles coluzzi* in West Africa is linked to increased expression of multiple gene families. BMC Genomics [Internet]. 2015;16:146 Available from: http://www.biomedcentral.com/1471-2164/16/146. 10.1186/s12864-015-1342-6 25766412 PMC4352231

[18] WHO. Test procedures for insecticide resistance monitoring in malaria vector mosquitoes. Geneva: World Health Organization; 2016; Available from: http://apps.who.int/iris/bitstream/10665/250677/1/9789241511575-eng.pdf.

[19] CDC. Guideline for evaluating insecticide resistance in vectors using the CDC bottle bioassay. Atlanta (GA): Centers for Disease Prevention and Control; 2013 Available from: https://www.cdc.gov/malaria/resources/pdf/fsp/ir_manual/ir_cdc_bioassay_en.pdf.

[20] Koama Bayili, Severin N’do, Moussa Namountougou, Roger Sanou, Ouattara Abdoulaye, Roch K. Dabiré et al Evaluation of efcacy of Interceptor^®^G2, a long-lasting insecticide net coated with a mixture of chlorfenapyr and alpha-cypermethrin, against pyrethroid resistant *Anopheles gambiae* s.l. in Burkina Faso. *Malar J* (2017) 16:190, 10.1186/s12936-017-1846-4 28482891 PMC5422893

[21] Protopopoff N, Mosha JF, Lukole E, Charlwood JD, Wright A, Mwalimu CD, et al Effectiveness of a long-lasting piperonyl butoxide-treated insecticidal net and indoor residual spray interventions, separately and together, against malaria transmitted by pyrethroid-resistant mosquitoes: a cluster, randomised controlled, two-by-two factorial design trial. Lancet 2018;391(10130):1577–1588. 10.1016/S0140-6736(18)30427-6 29655496 PMC5910376

[22] Bingham G, Strode C, Tran L, Khoa PT, Jamet HP. Can piperonyl butoxide enhance the efficacy of pyrethroids against pyrethroid-resistant *Aedes aegypti*? Trop Med Int Health. 2011;16:492–500. 10.1111/j.1365-3156.2010.02717.x 21324051

[23] Chouaïbou M, Zivanovic GB, Knox TB, Jamet HP, Bonfoh B. Synergist bioassays: a simple method for initial metabolic resistance investigation of field *Anopheles gambiae* s.l. populations. Acta Trop. 2014;130:108–111. 10.1016/j.actatropica.2013.10.020 24191946 PMC4786622

[24] Sarah G. Staedke, Moses R. Kamya, Grant Dorsey, Catherine Maiteki-Sebuguzi, Samuel Gonahasa, Adoke Yeka et al LLIN Evaluation in Uganda Project (LLINEUP) Impact of long-lasting insecticidal nets with, and without, piperonyl butoxide on malaria indicators in Uganda: study protocol for a cluster-randomised trial. Trials 2019, 10.1186/s13063-019-3382-8. PMC6547536 31159887

[25] Jacob M. Riveron, Silvie Huijben, Williams Tchapga, Magellan Tchouakui, Murielle J. Wondji, Micareme Tchoupo et al Escalation of pyrethroid resistance in the malaria vector *Anopheles funestus* induces a loss of efficacy of piperonyl butoxide–based insecticide-treated nets in Mozambique. Jour Infec Dis, 2019;220:467–75, 10.1093/infdis/jiz139 30923819 PMC6603977

[26] Gleave K, Lissenden N, Richardson M, Ranson H. Piperonyl butoxide (PBO) combined with pyrethroids in long-lasting insecticidal nets (LLINs) to prevent malaria in Africa. *Cochrane Database of Systematic Reviews* 2017, Issue 8 Art. No.: CD012776. 10.1002/14651858.CD012745 www.cochranelibrary.com PMC6262905 30488945

