# Supplementary material for: Experimental hut evaluation of DawaPlus 3.0 LN and DawaPlus 4.0 LN treated with deltamethrin and PBO against free-flying populations of Anopheles gambiae s.l. in Vallée du Kou, Burkina Faso
Source: PLoS One. 2019 Dec 23;14(12):e0226191. doi: 10.1371/journal.pone.0226191 (PMC6927612; doi:10.1371/journal.pone.0226191)
Supplement: S5 File — (PDF) [file pone.0226191.s005.pdf]

TEST REPORT

Report nr **RE / 17 / U10 / 24417 / 2** version **01**
**WHOPES Phase II testing and evaluation of DawaPlus 3.0 and DawaPlus 4.0 in Burkina Faso**
Number of appendix(ces) : 0 page(s)
Contact e-mail : o.pigeon@cra.wallonie.be

Walloon Agricultural Research Centre (CRA-W)
Agriculture and Natural Environment Department (D3)
Plant Protection Products and Biocides Physico-chemistry and Residues Unit (U10)
Carson Building
Rue du Bordia, 11
B-5030 Gembloux
BELGIUM
Phone : ++32 (0) 81 62 52 62
Fax : ++32 (0) 81 62 52 72
http://cra.wallonie.be

cra-w

BELAC 189-TEST

1. Report to the attention of

Company : World Health Organization (WHO) VAT Number :
WHO Pesticide Evaluation Scheme (WHOPES)
Street : Avenue Appia Nr : 20 Box :
Postal code : CH-1211 City : GENEVA 27 Country : SWITZERLAND
Contact person : Dr Rajpal YADAV E-mail : yadavraj@who.int
Mobile phone : Phone : +41 22 791 29 61 Fax : +41 22 791 31 11

2. Samples

Nature:
- **DawaPlus 3.0** [deltamethrin 2.5 g/kg (for 100 denier yarn) long-lasting (coated onto polyester) insecticidal net (LN)] for the sides combined with [deltamethin 3.0 g/kg + piperonyl butoxide 11 g/kg long-lasting (incorporated into polyethylene) insecticidal net (LN)] for the roof, as candidate LN
- **DawaPlus 4.0** [deltamethin 3.0 g/kg + piperonyl butoxide 11 g/kg long-lasting (incorporated into polyethylene) insecticidal net (LN)], as candidate LN
- **DawaPlus 2.0** [deltamethrin 2.0 g/kg (for 100 denier yarn) long-lasting (coated onto polyester) insecticidal net (LN)], as positive control
- **Untreated net**

Net samples sent by the IRSS / Centre Muraz, Burkina Faso for the **WHOPES Phase II testing and evaluation of DawaPlus 3.0 and DawaPlus 4.0 in Burkina Faso.**

Number:
- 2 batches : initial (before and after washing) and after field trial.
- 7 arms : unwashed DawaPlus 3.0, DawaPlus 3.0 washed 20 times, unwashed DawaPlus 4.0, DawaPlus 4.0 washed 20 times, unwashed DawaPlus 2.0, DawaPlus 2.0 washed 20 times, untreated net.
- 5 positions on the net (L1, L2, l1, l2, H = roof).

Total = 68 net samples (1 piece of 30 cm x 30 cm per net sample).

Receipt date : January 09, 2017

Pre-treatment/storage before analysis* : at room temperature in the original packaging under shelter from direct sunlight

Lab Id number : Mo 481/1 to Mo 481/68 Storage* : 3 months after analysis

* Irrelevant if not specified

3. Performed analyses

Analysis	Methods or standards	Sub-contracting (name)	Accredited ISO17025 (A)	Dates
Laboratory sampling	Cutting of a circular piece of 100 cm² from the net sample in order to determine fabric weight.<br>Cutting of the rest of the net sample into small pieces of 5-10 mm square and homogenization in order to determine deltamethrin and/or piperonyl butoxide content.			02/02/2017 to 08/02/2017

Fabric weight<br>(mass of net per m²)	CRA-W PA-U10-NET001<br>(1 determination per net sample).<br><br>Measurement of the weight of 1 circular piece of 100 cm² and expression of the result as g of net per m².			14/02/2017<br>to<br>16/02/2017
Deltamethrin content in DawaPlus 3.0 side panels and DawaPlus 2.0	CRA-W PA-U10-RESSM014<br><u>Based on</u><br>CIPAC 333/LN/(M)/3 (HPLC-DAD),<br>CIPAC Handbook M, page 66<br>(1 determination per net sample).<br><br>Extraction by sonication and shaking with isooctane/dioxane (80/20, v/v) in presence of dipropyl phthalate as internal standard and determination by High Performance Liquid Chromatography with UV Diode Array Detection (HPLC-DAD).		**(A)**	14/02/2017<br>to<br>22/02/2017
Deltamethrin content in DawaPlus 3.0 roof, DawaPlus 4.0 and Untreated net	CRA-W PA-U10-RESSM021<br><u>Based on</u><br>CIPAC 333/LN/(M2)/3 (HPLC-DAD),<br>CIPAC Handbook N, page 34<br>(1 determination per net sample).<br><br>Extraction by refluxing for 30 minutes with xylene in presence of dicyclohexyl phthalate as internal standard, solvent exchange to the mobile phase and determination by High Performance Liquid Chromatography with UV Diode Array Detection (HPLC-DAD).		**(A)**	16/02/2017<br>to<br>01/03/2017
Piperonyl butoxide content in DawaPlus 3.0 roof, DawaPlus 4.0 and Untreated polyester net	<u>For extraction</u><br>CRA-W PA-U10-RESSM021<br><u>Based on</u><br>CIPAC 333/LN/(M2)/3 (HPLC-DAD),<br>CIPAC Handbook N, page 34<br><u>For chromatographic determination</u><br>CRA-W PA-U10-RESSM026<br><u>Based on</u><br>CIPAC 33/LN/(M)/3 (GC-FID),<br>CIPAC Handbook N, page 112<br>(1 determination per net sample)<br>Extraction by refluxing for 30 minutes with xylene in presence of dicyclohexyl phthalate as internal standard and determination by Gas Chromatography using Flame Ionisation Detection (GC-FID).			16/02/2017<br>to<br>28/02/2017

4. Results

4.1 Deltamethrin content in DawaPlus 3.0, DawaPlus 4.0, DawaPlus 2.0 and Untreated net before and after washing (initial)

Net Type	Net piece location	Laboratory sample number	Deltamethrin content (g/kg)			Fabric weight (g/m²)			Deltamethrin content (mg/m²)		
			Individual result (*)	Mean	RSD (%)	Individual result	Mean	RSD (%)	Individual result (*)	Mean	RSD (%)
Control	1	Mo 481/1	**< 0.01**	**< 0.01**	-	38.0	-	-	< 0.4	< 0.4	-
Unwashed DawaPlus 2.0	L 2	Mo 481/7	**2.22**			41.4			92		
	I 1	Mo 481/8	**2.17**			41.4			90		
	I 2	Mo 481/9	**2.22**			39.8			88		
	H = Roof	Mo 481/10	**2.19**	**2.20**	**1.2%**	39.4	40.5	2.6%	86	89	2.6%
DawaPlus 2.0 washed 20 times	L 1	Mo 481/11	**0.74**			45.6			34		
	L 2	Mo 481/12	**0.76**			43.9			33		
	I 1	Mo 481/13	**0.78**			43.4			34		
	I 2	Mo 481/14	**0.81**			44.4			36		
	H = Roof	Mo 481/15	**0.76**	**0.77**	**3.1%**	43.3	44.1	2.1%	33	34	3.5%
Unwashed DawaPlus 3.0	L 2	Mo 481/16	**2.63**			39.7			104		
	I 1	Mo 481/17	**2.66**			39.0			104		
	I 2	Mo 481/18	**2.65**	**2.64**	**0.5%**	39.6	39.4	1.0%	105	104	0.6%
	H 1	Mo 481/19	**2.75**			42.1			116		
	H 2	Mo 481/20	**2.69**	**2.72**	**1.8%**	41.0	41.6	1.9%	110	113	3.7%
DawaPlus 3.0 washed 20 times	L 1	Mo 481/21	**0.18**			41.2			7		
	L 2	Mo 481/22	**0.17**			42.0			7		
	I 1	Mo 481/23	**0.30**			40.9			12		
	I 2	Mo 481/24	**0.24**	**0.22**	**27.7%**	41.2	41.3	1.1%	10	9	26.8%
	H = Roof	Mo 481/25	**2.43**	**-**	**-**	48.1	-	-	117	-	-
Unwashed DawaPlus 4.0	L 2	Mo 481/26	**3.23**			38.1			123		
	I 1	Mo 481/27	**3.14**			36.6			115		
	I 2	Mo 481/28	**3.04**			36.7			112		
	H = Roof	Mo 481/29	**3.05**	**3.11**	**2.8%**	38.0	37.3	2.2%	116	116	4.1%
DawaPlus 4.0 washed 20 times	L 1	Mo 481/30	**1.87**			49.5			93		
	L 2	Mo 481/31	**1.94**			47.0			91		
	I 1	Mo 481/32	**1.96**			46.0			90		
	I 2	Mo 481/33	**1.81**			50.2			91		
	H = Roof	Mo 481/34	**1.77**	**1.87**	**4.3%**	51.0	48.7	4.4%	90	91	1.2%

4.2 Deltamethrin content in DawaPlus 3.0, DawaPlus 4.0, DawaPlus 2.0 and Untreated net after field trial

Net Type	Net piece location	Laboratory sample number	Deltamethrin content (g/kg)			Fabric weight (g/m²)			Deltamethrin content (mg/m²)		
			Individual result (*)	Mean	RSD (%)	Individual result	Mean	RSD (%)	Individual result (*)	Mean	RSD (%)
Control	L 1	Mo 481/2	**< 0.01**			40.3			< 0.4		
	L 2	Mo 481/3	**< 0.01**			40.0			< 0.4		
	l 1	Mo 481/4	**< 0.01**			40.9			< 0.4		
	l 2	Mo 481/5	**< 0.01**			39.3			< 0.4		
	H = Roof	Mo 481/6	**< 0.01**	**< 0.01**	**-**	39.4	40.0	1.7%	< 0.4	< 0.4	-
Unwashed DawaPlus 2.0	L 1	Mo 481/35	**1.83**			42.9			78		
	L 2	Mo 481/36	**1.99**			50.3			100		
	l 1	Mo 481/37	**1.81**			42.4			77		
	l 2	Mo 481/38	**2.01**			41.0			82		
	H = Roof	Mo 481/39	**2.07**	**1.94**	**6.0%**	41.9	43.7	8.6%	87	85	11.1%
DawaPlus 2.0 washed 20 times	L 1	Mo 481/40	**0.71**			45.2			32		
	L 2	Mo 481/41	**0.84**			44.1			37		
	l 1	Mo 481/42	**0.81**			46.7			38		
	l 2	Mo 481/43	**0.84**			43.4			37		
	H = Roof	Mo 481/44	**0.75**	**0.79**	**7.4%**	44.2	44.7	2.8%	33	35	7.1%
Unwashed DawaPlus 3.0	L 1	Mo 481/45	**2.47**			39.5			98		
	L 2	Mo 481/46	**2.52**			38.5			97		
	l 1	Mo 481/47	**2.36**			42.9			101		
	l 2	Mo 481/48	**2.42**	**2.44**	**0.03**	40.5	40.3	4.7%	98	98	2.0%
	H1 = Roof	Mo 481/49	**2.67**			40.2			107		
	H2 = Roof	Mo 481/50	**2.51**			41.5			104		
	H3 = Roof	Mo 481/51	**2.63**	**2.60**	**3.3%**	40.4	40.7	1.8%	106	106	1.5%
DawaPlus 3.0 washed 20 times	L 1	Mo 481/52	**0.18**			42.0			8		
	L 2	Mo 481/53	**0.12**			42.6			5		
	l 1	Mo 481/54	**0.20**			46.0			9		
	l 2	Mo 481/55	**0.24**	**0.19**	**0.26**	42.6	43.3	4.2%	10	8	27.2%
	H1 = Roof	Mo 481/56	**2.87**			46.5			133		
	H2 = Roof	Mo 481/57	**3.07**			46.6			143		
	H3 = Roof	Mo 481/58	**2.99**	**2.97**	**3.4%**	46.9	46.7	0.4%	140	139	3.5%
Unwashed DawaPlus 4.0	L 1	Mo 481/59	**2.44**			39.1			96		
	L 2	Mo 481/60	**2.54**			39.3			100		
	l 1	Mo 481/61	**2.43**			42.7			104		
	l 2	Mo 481/62	**2.46**			39.4			97		
	H = Roof	Mo 481/63	**2.41**	**2.46**	**2.0%**	38.2	39.8	4.3%	92	98	4.4%
DawaPlus 4.0 washed 20 times	L 1	Mo 481/64	**1.97**			46.1			91		
	L 2	Mo 481/65	**2.29**			45.8			105		
	l 1	Mo 481/66	**2.07**			46.1			95		
	l 2	Mo 481/67	**2.38**			44.4			106		
	H = Roof	Mo 481/68	**1.98**	**2.14**	**8.8%**	45.5	45.5	1.5%	90	97	7.7%

(*) Each result is the mean of 2 chromatographic injections (duplicate injections).
LOQ = limit of quantification = 0.01 g/kg.
U = expanded uncertainty = 0.06 g/kg at 1.3 g/kg for DawaPlus 3.0 side panels and DawaPlus 2.0.
U = expanded uncertainty = 0.25 g/kg at 1.7 g/kg for DawaPlus 3.0 roof, DawaPlus 4.0 and Untreated nets.

For DawaPlus 3.0 side panels and DawaPlus 2.0 (coated LNs), the replicate analysis (n = 2, RSD = 1.4%) of a quality control sample performed concurrently with the analysis of net samples showed results within the limits of the quality control chart. The good accuracy and reproducibility of the analytical method are therefore confirmed.

For DawaPlus 3.0 roof, DawaPlus 4.0 (incorporated LNs) and Untreated nets, the replicate analysis (n = 2, RSD = 0.6%) of a quality control sample performed concurrently with the analysis of net samples showed results within the limits of the quality control chart. The good accuracy and reproducibility of the analytical method are therefore confirmed.

Deltamethrin *R*-alpha isomer [α*R*,1*R*,3*R*-isomer] is a non-relevant impurity of Deltamethrin [α*S*,1*R*,3*R*-isomer]. This isomer is not part of the active ingredient and has no insecticidal activity. For DawaPlus 3.0 side panels and DawaPlus 2.0 (coated LNs), the deltamethrin *R*-alpha isomer content was less than 0.03 g/kg corresponding to less than 1.5% of the deltamethrin content. For DawaPlus 3.0 roof and DawaPlus 4.0 (incorporated LNs), the deltamethrin *R*-alpha isomer content was less than 0.08 g/kg corresponding to less than 3% of the deltamethrin content.

4.3 Piperonyl butoxide content in DawaPlus 3.0 roof, DawaPlus 4.0 and Untreated net before and after washing (initial)

Net Type	Net piece location	Laboratory sample number	Piperonyl butoxide content (g/kg)			Fabric weight (g/m²)			Piperonyl butoxide content (mg/m²)		
			Individual result (*)	Mean	RSD (%)	Individual result	Mean	RSD (%)	Individual result (*)	Mean	RSD (%)
Control	1	Mo 481/1	**< 0.1**	**-**	**-**	38.0	-	-	< 4	< 4	-
Unwashed DawaPlus 2.0	L 2	Mo 481/7	**-**			-			-		
	l 1	Mo 481/8	**-**			-			-		
	l 2	Mo 481/9	**-**			-			-		
	H = Roof	Mo 481/10	**-**	**-**	**-**	**-**	**-**	**-**	**-**	**-**	**-**
DawaPlus 2.0 washed 20 times	L 1	Mo 481/11	**-**			-			-		
	L 2	Mo 481/12	**-**			-			-		
	l 1	Mo 481/13	**-**			-			-		
	l 2	Mo 481/14	**-**			-			-		
	H = Roof	Mo 481/15	**-**	**-**	**-**	-	-	-	-	-	-
Unwashed DawaPlus 3.0	L 2	Mo 481/16	**-**						-		
	l 1	Mo 481/17	**-**						-		
	l 2	Mo 481/18	**-**						-		
	H 1	Mo 481/19	**8.4**			42.1			356		
	H 2	Mo 481/20	**9.1**	**8.7**	**5%**	41.0	41.6	1.9%	371	364	3%
DawaPlus 3.0 washed 20 times	L 1	Mo 481/21	**-**			**-**			**-**		
	L 2	Mo 481/22	**-**			**-**			**-**		
	l 1	Mo 481/23	**-**			-			**-**		
	l 2	Mo 481/24	**-**			-			**-**		
	H = Roof	Mo 481/25	**5.7**	**-**	**-**	48.1	**-**	**-**	272	**-**	**-**
Unwashed DawaPlus 4.0	L 2	Mo 481/26	**10.2**			38.1			390		
	l 1	Mo 481/27	**10.4**			36.6			380		
	l 2	Mo 481/28	**10.9**			36.7			398		
	H = Roof	Mo 481/29	**10.3**	**10.4**	**3%**	38.0	37.3	2.2%	390	390	2%
DawaPlus 4.0 washed 20 times	L 1	Mo 481/30	**1.9**			49.5			95		
	L 2	Mo 481/31	**2.6**			47.0			124		
	l 1	Mo 481/32	**3.1**			46.0			142		
	l 2	Mo 481/33	**2.2**			50.2			111		
	H = Roof	Mo 481/34	**2.3**	**2.4**	**18%**	51.0	48.7	4.4%	119	118	15%

4.4 Piperonyl butoxide content in DawaPlus 3.0 roof, DawaPlus 4.0 and Untreated net after field trial

Net Type	Net piece location	Laboratory sample number	Piperonyl butoxide content (g/kg)			Fabric weight (g/m²)			Piperonyl butoxide content (mg/m²)		
			Individual result (*)	Mean	RSD (%)	Individual result	Mean	RSD (%)	Individual result (*)	Mean	RSD (%)
Control	L 1	Mo 481/2	**< 0.1**			40.3			< 4		
	L 2	Mo 481/3	**< 0.1**			40.0			< 4		
	l 1	Mo 481/4	**< 0.1**			40.9			< 4		
	l 2	Mo 481/5	**< 0.1**			39.3			< 4		
	H = Roof	Mo 481/6	**< 0.1**	**< 0.1**	**-**	39.4	40.0	1.7%	< 4	< 4	-
Unwashed DawaPlus 2.0	L 1	Mo 481/35	-			-			-		
	L 2	Mo 481/36	-			-			-		
	l 1	Mo 481/37	-			-			-		
	l 2	Mo 481/38	-			-			-		
	H = Roof	Mo 481/39	-	-	-	-	-	-	-	-	-
DawaPlus 2.0 washed 20 times	L 1	Mo 481/40	-			-			-		
	L 2	Mo 481/41	-			-			-		
	l 1	Mo 481/42	-			-			-		
	l 2	Mo 481/43	-			-			-		
	H = Roof	Mo 481/44	-	-	-	-	-	-	-	-	-
Unwashed DawaPlus 3.0	L 1	Mo 481/45	-			-			-		
	L 2	Mo 481/46	-			-			-		
	l 1	Mo 481/47	-			-			-		
	l 2	Mo 481/48	-			-			-		
	H1 = Roof	Mo 481/49	**6.6**			40.2			265		
	H2 = Roof	Mo 481/50	**7.2**			41.5			298		
	H3 = Roof	Mo 481/51	**6.9**	**6.9**	**4%**	40.4	40.7	1.8%	279	281	6%
DawaPlus 3.0 washed 20 times	L 1	Mo 481/52	-			-			-		
	L 2	Mo 481/53	-			-			-		
	l 1	Mo 481/54	-			-			-		
	l 2	Mo 481/55	-			-			-		
	H1 = Roof	Mo 481/56	**4.2**			46.5			197		
	H2 = Roof	Mo 481/57	**4.2**			46.6			197		
	H3 = Roof	Mo 481/58	**4.3**	**4.3**	**1%**	46.9	46.7	0.4%	203	199	2%
Unwashed DawaPlus 4.0	L 1	Mo 481/59	**9.1**			39.1			358		
	L 2	Mo 481/60	**9.2**			39.3			362		
	l 1	Mo 481/61	**9.2**			42.7			392		
	l 2	Mo 481/62	**9.1**			39.4			360		
	H = Roof	Mo 481/63	**9.0**	**9.1**	**1%**	38.2	39.8	4.3%	343	363	5%
DawaPlus 4.0 washed 20 times	L 1	Mo 481/64	**1.5**			46.1			69		
	L 2	Mo 481/65	**2.1**			45.8			94		
	l 1	Mo 481/66	**1.9**			46.1			88		
	l 2	Mo 481/67	**2.0**			44.4			88		
	H = Roof	Mo 481/68	**1.7**	**1.8**	**12%**	45.5	45.5	1.5%	78	84	12%

(*) Each result is the mean of 2 chromatographic injections (duplicate injections).
LOQ = limit of quantification = 0.1 g/kg.

4.5 Summary of results for deltamethrin

Target dose and tolerance limit for deltamethrin content in baseline DawaPlus 3.0 side panels 100 denier = 2.5 g/kg ± 25% for 100 denier yarn [1.9 g/kg - 3.1 g/kg] according to the draft specification provided by the manufacturer to WHO.

Target dose and tolerance limit for deltamethrin content in baseline DawaPlus 3.0 roof and DawaPlus 4.0 = 3.0 g/kg ± 25% [2.3 g/kg - 3.8 g/kg] according to the draft specification provided by the manufacturer to WHO.

Target dose and tolerance limit for deltamethrin content in baseline DawaPlus 2.0 = 2.0 g/kg ± 25% for 100 denier yarn [1.5 g/kg - 2.5 g/kg] according to WHO specification 333/LN/2 (December 2014).

The tolerances refer to the average analytical result obtained and take into account manufacturing, sampling and analytical variations. The sample will be considered to comply with the specification if the average analytical result lies within the tolerance range of the declared content.

Treatment	Burkina Faso			
	AI content (g/kg) before washing	AI content (g/kg) after 20 washes	AI retention (% of wash 0)	AI content (g/kg) after field trial
Unwashed DawaPlus 3.0 roof	2.72	-	-	2.60
DawaPlus 3.0 roof washed 20 times	-	2.43	89%	2.97
Unwashed DawaPlus 3.0 sides	2.64	-	-	2.44
DawaPlus 3.0 sides washed 20 times	-	0.22	8%	0.19
Unwashed DawaPlus 4.0	3.11	-	-	2.46
DawaPlus 4.0 washed 20 times	-	1.87	60%	2.14
Unwashed DawaPlus 2.0	2.20	-	-	1.94
DawaPlus 2.0 washed 20 times	-	0.77	35%	0.79
Untreated polyester net	< 0.01	-	-	< 0.01

Compliance of baseline samples with WHO specifications

LN	Burkina Faso		
	AI content (g/kg)	Compliance with specification	AI within-net variation (RSD)
DawaPlus 3.0 roof - 0 wash - BW	2.72	Yes	1.8%
DawaPlus 3.0 sides - 0 wash - BW	2.64	Yes	0.5%
DawaPlus 4.0 - 0 wash - BW	3.11	Yes	2.8%
DawaPlus 2.0 - 0 wash - BW	2.20	Yes	1.2%

4.6 Summary of results for piperonyl butoxide

Target dose and tolerance limit for piperonyl butoxide content in baseline DawaPlus 3.0 roof and DawaPlus 4.0 = 11.0 g/kg ± 25% [8.3 g/kg - 13.8 g/kg] according to the draft specification provided by the manufacturer to WHO.

**Treatment**	**Burkina Faso**			
	AI content (g/kg) before washing	AI content (g/kg) after 20 washes	AI retention (% of wash 0)	AI content (g/kg) after field trial
Unwashed DawaPlus 3.0 roof	8.7	-	-	6.9
DawaPlus 3.0 roof washed 20 times	-	5.7	65%	4.3
Unwashed DawaPlus 4.0	10.4	-	-	9.1
DawaPlus 4.0 washed 20 times	-	2.4	23%	1.8
Untreated net	< 0.1	-	-	< 0.1

<u>Compliance of baseline samples with WHO specification</u>

**LN**	**Burkina Faso**		
	AI content (g/kg)	Compliance with specification	AI within-net variation (RSD)
DawaPlus 3.0 roof - 0 wash - BW	8.7	Yes	5%
DawaPlus 4.0 - 0 wash - BW	10.4	Yes	3%

5. Opinions, interpretations and advices **

The analytical method used for determination of deltamethrin in samples of **DawaPlus 3.0 side panels** and **DawaPlus 2.0** is based on the CIPAC method 333/LN/(M)/3. This method involves extraction of deltamethrin by sonication and shaking with isooctane / dioxane (80/20, v/v) in presence of dipropyl phthalate as internal standard and determination by High Performance Liquid Chromatography with UV Diode Array Detection (HPLC-DAD). The performance of the analytical method was controlled during the analysis of samples in order to validate the analytical results.

The analytical method used for determination of deltamethrin in samples of **DawaPlus 3.0 roof**, **DawaPlus 4.0** and **Untreated nets** is based on the CIPAC method 333/LN/(M2)/3. This method involves extraction of deltamethrin by refluxing for 30 minutes with xylene in presence of dicyclohexyl phthalate as internal standard, solvent exchange to the mobile phase and determination by High Performance Liquid Chromatography with UV Diode Array Detection (HPLC-DAD). The performance of the analytical method was controlled during the analysis of samples in order to validate the analytical results.

The analytical method used for determination of piperonyl butoxide in samples of **DawaPlus 3.0 roof, DawaPlus 4.0** and **Untreated nets** is based on the CIPAC methods 333/LN/(M2)/3 for the extraction and 33/LN/(M)/3 for the chromatographic determination. This method involves extraction of piperonyl butoxide by refluxing for 30 minutes with xylene in presence of dicyclohexyl phthalate as internal standard and determination by Gas Chromatography with Flame Ionisation Detection (GC-FID). The performance of the analytical method was controlled during the analysis of samples in order to validate the analytical results.

The mean deltamethrin content in 1 unwashed **DawaPlus 3.0 side panels** is 2.64 g/kg. The net comply with the target dose of 2.5 g/kg ± 25% [1.9 - 3.1 g/kg]. The within-net variation, expressed as the relative standard deviation (RSD) of the deltamethrin content found on 3 different pieces cut from the unwashed net is 0.5%, showing an acceptable homogeneity of the distribution of the active substance within the net. The deltamethin content is 0.22 g/kg after 20 washes, corresponding to an overall deltamethrin retention of 8%. After field trial, the deltamerthrin content does not decrease, as it is 2.44 g/kg for the unwashed DawaPlus 3.0 side panels and 2.97 g/kg for the DawaPlus 3.0 side panels washed 20 times.

The deltamethrin content in 1 unwashed **DawaPlus 3.0 roof** is 2.72 g/kg. The net comply with the target dose of 3.0 g/kg ± 25% [2.3 - 3.8 g/kg]. The within-net variation, expressed as the relative standard deviation (RSD) of the deltamethrin content found on 2 different pieces cut from the unwashed nets is 1.8%, showing an acceptable homogeneity of the distribution of the active substance within the net. The mean deltamethrin content in 1 unwashed **DawaPlus 4.0** is 3.11 g/kg. The net comply with the target dose of 3.0 g/kg ± 25% [2.3 - 3.8 g/kg]. The within-net variation, expressed as the relative standard deviation (RSD) of the deltamethrin content found on 5 different pieces cut from the unwashed net is 2.8%, showing an acceptable homogeneity of the distribution of the active substance within the net. The deltamethrin content is 1.87 g/kg after 20 washes, corresponding to an overall deltamethrin retention of 60%. After field trial, the deltamethrin content does not decrease, as it is 2.46 g/kg for the unwashed DawaPlus 4.0 and 2.14 g/kg for the DawaPlus 4.0 washed 20 times.

The mean deltamethrin content in 1 unwashed **DawaPlus 2.0** is 2.20 g/kg. The net comply with the target dose of 2.0 g/kg ± 25% [1.5 - 2.5 g/kg]. The within-net variation, expressed as the relative standard deviation (RSD) of the deltamethrin content found on 5 different pieces cut from the unwashed nets is 1.2%, showing an acceptable homogeneity of the distribution of the active substance within the net. The deltamethin content is 0.77 g/kg after 20 washes, corresponding to an overall deltamethrin retention of 35%. After field trial, the deltamethrin content does not decrease, as it is 1.94 g/kg for the unwashed DawaPlus 2.0 and 0.79 g/kg for the DawaPlus 2.0 washed 20 times.

The piperonyl butoxide content in 1 unwashed **DawaPlus 3.0 roof** is 8.7 g/kg. The net comply with the target dose of 11.0 g/kg ± 25% [8.3 - 13.8 g/kg]. The within-net variation, expressed as the relative standard deviation (RSD) of the piperonyl butoxide content found on 2 different pieces cut from the unwashed net is 5%, showing an acceptable homogeneity of the distribution of the synergist within the net. The mean piperonyl butoxide content in 1 unwashed **DawaPlus 4.0** is 10.4 g/kg. The net comply with the target dose of 11.0 g/kg ± 25% [8.3 - 13.8 g/kg]. The within-net variation, expressed as the relative standard deviation (RSD) of the piperonyl butoxide content found on 5 different pieces cut from the unwashed net is 3%, showing an acceptable homogeneity of the distribution of the synergist within the net. The piperonyl butoxide content is 2.4 g/kg after 20 washes, corresponding to an overall piperonyl butoxide retention of 23%. After field trial, the piperonyl butoxide content does not decrease, as it is 9.1 g/kg for the unwashed DawaPlus 4.0 and 1.8 g/kg for the DawaPlus 4.0 washed 20 times.

The deltamethrin and piperonyl butoxide content in the **Untreated net** before washing and after field trial is lower than the limit of quantification (< 0.01 g/kg for deltamethrin and < 0.1 g/kg for piperonyl butoxide).

** out of accreditation

6. Remark

WHO Purchase Order 21676728, WHO Reference 2017/695499-0

Confidentiality: the laboratory sees that appropriate measures are taken to ensure the confidentiality of the results. However unless otherwise stated (cf. contract), these results may be used anonymously for scientific purposes (including publication or oral communication).

Object: results given in this report are limited to the sample object(s) mentioned above. This report is in no way an approval of the analyzed product.

Reproduction: partial reproduction of the report is forbidden without written authorization of the laboratory.

Original: without the signature of the Laboratory manager, this report has to be considered as provisional.

Archiving: the test results are archived for 5 years.

Accreditation: a copy of the accreditation certificate and its technical annex as well as the uncertainty of the measurement for quantitative accredited method(s), the limit of detection (LOD) and quantification (LOQ) can be available on request to the Laboratory manager.

Contact: complaints or additional requests for information may be addressed per phone or in written form to the Laboratory manager.

Transmission of the draft report to the client: by ☐ fax ☐ e-mail ☒ none dated on ....

For approval:

The Laboratory Manager
Name: Dr ir Olivier PIGEON
Signature:

cra-w

Date : March 15, 2017
